# A German Book on Hospital Construction

**Published:** 1913-02-15

**Authors:** 


### A GERMAN BOOK ON HOSPITAL CONSTRUCTION.

One of the most instructive and authoritative works on hospital construction and management is that published two years ago by the well-known firm of Fischer of Jena. It is "Das Deutsche Krankenhaus: Handbuch für Bau, Einrichtung und Betrieb der Krankenanstalten" ("Handbook of Construction, Equipment, and Management of Hospitals"), and is issued under the joint editorship of Professor Dietrich, Chief Medical Officer of the German Ministry of Home Affairs, and Professor Dr. Grober, Director-General of the Hospitals in Essen. With them as co-workers are associated such well-known authorities as Architect Körner, Dr. Volhard of Mannheim, Dr. Kissling of Hamburg, Architect Böthke of Berlin, Superintendent Naumann, Professor Perthes of Tubingen, and Superintendent Pütter of Berlin. The book is a sumptuous monograph of more than a thousand pages, admirably illustrated, and giving concise bibliographies under the various sections. Such a work cannot be neglected by hospital workers in this country, and we therefore propose in this article to give some idea of its tenor and contents.

The opening section deals solely with construction. In the first two chapters Dr. Grober discusses the question of site and planning generally. He insists that no modern hospital should be built by one man; architect and medical man must work in conjunction, and both must give due regard to the interests, often widely conflicting, of those who have ultimately to work in the institution. He adds that there are few examples of ideal co-operation extant, but where these ideals have been realised the results have fully justified the pains taken to achieve ideal conditions. Many plans of new hospitals are given, together with some old ground plans to show where errors have been made and how they may be avoided in future. This part of the book appeals primarily to the architect, but it must by no means be neglected by the medical man, for it gives instructive suggestions and examples. Nor must we neglect the short chapter contributed by Architect Körner, who discusses the artistic side of hospital construction. This is an aspect, unfortunately, too often neglected by hospital constructors. Herr Körner shows how the planner of an ideal hospital must take into consideration many interests with regard to environment and decoration, and how these interests may be adequately considered without undue extravagance or architectonic redundancy. The next three exhaustive chapters are all by architects who are themselves responsible for much of the best work in hospital construction in Germany. Herr Böthke deals with details of building so far as the wards are concerned, Herr Guckuck with the economic blocks, and Herr Dietz with the technical details of ventilation and drainage. The plans given in this section are all well chosen and illustrate several important points. We may refer, for example, to the Altena Hospital, the Linden Hanover Hospital and the new Catholic Hospital at Kirchhellen, which give interesting details regarding the situation and planning of the economic blocks under certain difficulties. Specially interesting are the sections dealing with the control of hospital energy, by which is meant the arrangements for electrical, mechanical, steam, or other power used by the institution. The schematic diagram given on page 261 is admirably designed to show at a glance how these various sources may be controlled. The section is illustrated with numerous well-reproduced photographs which are elucidative and add greatly to the interest of the text.

The middle section deals with equipment, and is the joint work of several of the ablest hospital superintendents in the empire. We have space only to allude to the special articles on the construction and equipment of the laboratory and hydrotherapeutic blocks and to the clearly expressed chapter on the operating theatre. All these contain many suggestions and should be carefully studied. One of the most interesting is the chapter dealing with the mortuary unit which lays stress on the points we have alluded to in our special articles on this block now running in The Hospital. Another interesting chapter deals with wards and institutions for mental patients, another with military hospitals, and a third with the economic block. A feature of this part of the work is that full details are given of the equipment actually used—a most helpful point to hospital workers who desire to get comparative knowledge regarding the various instruments and furniture used in foreign hospitals. Plans and diagrams are always given and are generally instructive, though a few old blocks have been included in this section.

The third part of the book deals with management, and is the work also of hospital superintendents. Here one notices a certain amount of prolixity and repetition, but, on the whole, the chapters have been excellently written, and contain much original information which will be fully appreciated by the expert. Excerpt pages from the various day-books are given, and special attention is paid to the nursing section; to this section is appended a useful statistical table which gives details of the nursing staffs at various big general hospitals. It must be recognised that the percentage of nurses to patients is uniformly lower in Germany than it is with us; for the arguments in favour of such under-staffing, as some of us might call it, we must refer the reader to the article itself, which discusses the matter in detail. Dr. Pütter, the Director of the new Berlin Charité, deals with the question of secretarial management and discusses the various points in connection with the relation between the staff and the administration. His chapter is a very useful and instructive summary of what may be taken to be the prevailing Continental opinion, and gives interesting details regarding the financial control of the larger German institutions. Herr Naumann deals with the administration of the economic blocks. English workers will be interested in his schedule of diet qualities which are given in grammes and arranged in tabulated form so as to simplify reference. Very full lists are given in connection with the laundry work, and this part of the chapter contains some interesting illustrations showing how the soiled linen is dealt with at the big Hamburg hospitals.

Finally, we come to what is perhaps the most important of all the sections, that dealing with finance. In this full statistics are given regarding the building and administrative costs of some forty of the largest and best modern German hospitals, none of them more than ten years old and some of them only built two or three years ago. To the architect and superintendent and to all concerned in hospital construction this statistical table is both instructive and valuable, for it shows how the cost per bed has progressively risen during the last ten years. This rise, as we have frequently pointed out, is the result not of one isolated factor, but of many, and these factors are discussed at some length by Herr Pütter in his article. A very suggestive page is devoted to a discussion of new methods of cheaper building, notably the Dosquet pavilion system which has been adopted at Charlottenburg. Facsimiles of the ledgers and account books of large hospitals are given, which are interesting for purposes of comparison with our own uniform system of accounts.

Appended to this section are a couple of highly interesting articles contributed by Professor Dr. Zitelmann, of Bonn, on the legal questions in connection with the administration of hospitals. This is a very technical résumé, but is comparative in so far as the legal questions connected with English hospitals are also discussed. The final chapter deals with the official regulations regarding the building and administration of hospitals in the various German principalities and provinces.

This brief synopsis of the book will show the reader how exhaustive and thorough the whole work may claim to be. No single review can adequately condense the many interesting points which are dealt with, and we give this sketch merely to show our readers that Dr. Grober's book is one which should be in every institutional library. We have nothing precisely like it in English, and so far as Continental expert literature is concerned, it is the most up-to-date and authoritative handbook on the subject with which it deals.

